# Family Caregiver Acceptability of Assessing Caregiver Adverse Childhood Experiences (ACEs) and Distress in Pediatric Specialty Care

**DOI:** 10.3390/children10020382

**Published:** 2023-02-15

**Authors:** Theresa L. Kapke, Jeffrey Karst, Brynn LiaBraaten, Jian Zhang, Ke Yan, Jody Barbeau, Keri R. Hainsworth

**Affiliations:** 1 Department of Anesthesiology, Medical College of Wisconsin, Milwaukee, WI 53226, USA; 2 Jane B. Pettit Pain and Headache Center, Children’s Wisconsin, Wauwatosa, WI 53226, USA; 3 Division of Pediatric Psychology & Developmental Medicine, Department of Pediatrics, Medical College of Wisconsin, Milwaukee, WI 53226, USA; 4 MACC Fund Center for Cancer and Blood Disorders, Children’s Wisconsin, Wauwatosa, WI 53226, USA; 5 Division of Quantitative Health Sciences, Department of Pediatrics, Medical College of Wisconsin, Milwaukee, WI 53226, USA

**Keywords:** ACEs, resilience, caregiver wellbeing, pediatrics

## Abstract

Introduction: Providing quality healthcare for children includes assessing and responding to needs of their family caregivers. Three salient domains to consider are caregivers’ early adverse childhood experiences (ACEs), their current levels of distress, and their resilience in coping with both prior and current stressors. Objective: Determine acceptability of assessing caregiver ACEs, current distress, and resilience in pediatric subspecialty care settings. Methods: Caregivers of patients in two pediatric specialty care clinics completed questionnaires about their ACEs, recent emotional distress, and resilience. Importantly, caregivers also rated the acceptability of being asked these questions. Participants included 100 caregivers of youth ages 3–17 across Sickle Cell Disease and Pain clinic settings. The majority of participants were mothers (91.0%) who identified as non-Hispanic (86.0%). Caregiver race was primarily African American/Black (53.0%) and White (41.0%). The Area Deprivation Index (ADI) was used to assess socioeconomic disadvantage. Results: High levels of caregiver acceptability or neutrality with assessing ACEs and distress, as well as high ACEs, distress, and resilience. Associations were found between caregiver ratings of acceptability with caregiver resilience and socioeconomic disadvantage. Caregivers reported openness to being asked about their experiences during childhood and recent emotional distress, although ratings of acceptability varied according to other contextual variables, such as level of socioeconomic disadvantage and caregiver resilience. In general, caregivers perceived themselves to be resilient in the face of adversity. Conclusions: Assessing caregiver ACEs and distress in a trauma-informed way may provide opportunities for better understanding the needs of caregivers and families in order to support them more effectively in the pediatric setting.

## 1. Introduction

Early childhood adversity is detrimental for physical and psychosocial health. The American Academy of Pediatrics (AAP) has called for the integration of trauma-informed care to help mitigate the effects of early adversity for youth and families [1]. Providing trauma-informed care in a pediatric healthcare setting requires understanding both the experiences of the identified (pediatric) patient, as well as those of their family caregivers (including parents, grandparents, or other individuals who have provided consistent care for the child). Further, assessing caregiver functioning should include both assessment of current functioning and prior experiences. This perspective is supported by a significant body of research identifying relationships between caregiver psychosocial distress and higher levels of child pain [2,3], greater levels of inflammation [4], and poorer overall health status [5,6].

There are many domains of caregiver functioning worth consideration to best understand the current and prior experiences of caregivers. Particularly salient aspects of caregiver well-being include current distress, prior traumatic experiences, and resilience as a moderating variable that could potentially mitigate the impact of parent experiences on their child’s well-being. Current distress of parents in a pediatric setting should be specifically assessed in the context of caring for a chronically ill child, including assessment of both practical or functional concerns as well as emotional or social challenges [7]. Caregiver distress is particularly important to assess given a wealth of research suggesting that parents and caregivers of children with chronic illness experience heightened stress [8] and poorer overall mental and physical health [9]. Furthermore, caregiver distress reciprocally impacts the child and their ability to safely and effectively access healthcare (e.g., [10]). Therefore, assessing distress would allow providers to identify family level barriers that may be addressed with psychosocial services such as social work or behavioral health providers. 

In addition, understanding the prior traumatic experiences of caregivers appears essential given the broad impact that adverse childhood experiences have on physical and emotional health (ACEs; [11,12]). Assessment of caregivers’ ACEs allows for understanding of experiences ranging from psychological and physical to instances of sexual abuse, mental illness, and incarceration, that may have a significant and lasting impact on the mental and physical well-being of caregivers. These events are quite common, as a large study of ACEs [12] suggested that half of respondents experienced at least one ACE, with 25% reporting two or more. Importantly, research suggests that parent ACEs directly impact children, leading to higher levels of adversity and vulnerability [13]. Further, research suggests that higher levels of maternal ACEs are linked to increased non-adherence in pediatric medical settings [14]. It stands to reason that a large proportion of parents surveyed in a pediatric health setting may have salient ACEs to report, and if assessed, it would allow providers to better understand the physical and emotional health risks of both the caregiver and their child.

In addition to assessing prior experiences and current distress, assessing caregiver resilience provides the opportunity to understand coping strategies that may help caregivers address their own stressors, potentially mitigating the reciprocal impact on their child [15]. Resilience can be understood as both a process and an outcome, as the ability of an individual or family to adapt to their circumstances using both internal and external resources or as a multidimensional construct that summarizes positive outcomes in spite of challenging circumstances [16]. Despite the increased stressors faced by parents of children with chronic illness, research has also identified this group as resilient [17] in the face of these demands. Better understanding specific resilience factors that caregivers possess may also help providers tailor guidance to more effectively help families cope with the adversity and stress associated with managing their child’s illness [16].

Through assessment of both current and prior caregiver distress and resilience factors that may impact the manifestation of this distress, healthcare providers may be able to better support patients and families in a meaningful way. “Compassionate surveillance” refers to the process of working to identify individuals who have been negatively impacted by trauma and other social determinants of health (SDOHs) in order to connect families to necessary resources and supports. Compassionate surveillance often involves the use of standardized questions to assess for risk and protective factors in addition to clinical interviews and behavioral observations. Current guidelines for best practice encourage this kind of screening to be conducted within established provider–family relationships and trauma-informed settings [1]. Despite the call for compassionate surveillance, there is ongoing concern about screening for such in pediatrics due to worries about (1) the questions being perceived as intrusive, (2) causing unnecessary or significant distress for the child and family and/or additional burden for providers and staff, and (3) having limited psychosocial supports available to respond to any distress or experiences endorsed by families [18,19,20,21].

The concerns that have been raised are valid, especially across pediatric settings with limited resources and infrastructure to support such efforts. However, research demonstrates that when screening is done in a supportive manner, families appear to respond favorably, and providers have found it to be feasible to conduct such surveillance and screening in pediatric settings [1,18]. Additionally, recommendations have been provided about how to screen for SDOHs in a way that does not lead to unintended negative consequences for youth and families, including universal, patient- and family-centered screenings, clinical response teams that support referrals to community-based resources, and other initiatives to promote resilience in youth and families [19].

Although some clinics and healthcare systems have begun to screen for parental ACEs and related factors in the primary care setting, e.g., [20], less is known about screening in the pediatric specialty clinic setting. Given the potential barriers associated with compassionate surveillance and paucity of research within specialty clinics, it is important that we examine caregiver responses in this setting where families may receive targeted care that allows for closer and well-established provider–caregiver relationships. 

The current study examined caregiver ratings of acceptability related to assessing caregiver ACEs and recent emotional distress, as well as caregiver resilience amongst caregivers of youth with Sickle Cell Disease (SCD) or chronic pain. These patient populations were included in the current study as research suggests that compared to community-based samples, families of youth with SCD or chronic pain demonstrate higher rates of ACEs and psychosocial stressors [22,23]. The objective of this study was to understand the feasibility and acceptability of assessing distress, ACEs, and resilience in pediatric subspecialty care settings. It was hypothesized that caregivers would report high acceptability for screening, as well as high ACEs, recent distress, and resilience. Based on previous research that found high levels of acceptability of SDOHs screening amongst diverse youth and families [24,25], it was also hypothesized that families with greater socioeconomic disadvantage would report higher ratings of acceptability related to assessing caregiver ACEs and recent distress. 

## 2. Materials and Methods

The data were collected as part of a larger study focused on caregiver and child health. This study was approved by the hospital Institutional Review Board. Data are available upon request.

Convenience sampling across two pediatric subspecialty clinics (one for Chronic Pain and one for Sickle Cell Disease) was used to enroll participants, with a goal of 50 enrolled participants in each cohort. The sample size was derived via power analysis for subsequent examination of the relationship of caregiver distress, resilience, and ACEs with healthcare utilization (not included in this paper). Participants included 100 caregivers of youth between the ages of 3 and 17 years, primarily mothers (91.0%; Table 1). 

Caregiver ethnicity and race were primarily non-Hispanic (86.0%) and African American or Black (53.0%) or White (41.0%). To participate in the current study, caregivers had to be English-speaking, legal guardians of youth seeking care for SCD or chronic pain across two different pediatric specialty clinic settings. Families were invited to participate by a member of the research team during one of their child’s medical visits. Families were informed that their decision to participate in the current study would not impact their medical care in any way. Informed consent was obtained for all participants, and caregivers completed questionnaires on tablets in private following their child’s medical visit. All participants were compensated for their time and provided with stress management and mental health resources.

Caregivers completed a series of self-report measures, including a demographic questionnaire, a 14-item caregiver ACE measure [12,20,26]; the 11-point Distress Thermometer [27], and the 9-point Connor–Davidson Resilience Scale [CD-RISC 2, [28]]. Higher scores on the NCCN and CD-RISC 2 indicated higher levels of recent emotional distress and resilience. Separate questions were used to assess acceptability of asking about one’s (1) experiences during childhood and (2) recent emotional distress. Both questions used the same stem: “On a scale of 1 to 5, how acceptable do you think it was for us to ask you about your (1) childhood experiences, (2) recent emotional distress?” (anchors: 1 = extremely unacceptable, 3 = neutral, and 5 = extremely acceptable). The Area Deprivation Index (ADI) was used to assess level of socioeconomic disadvantage on the state level based on family’s residential zip code with higher scores indicating greater socioeconomic disadvantage [29,30]. 

The 14-item ACE measure that was used in the current study had been modified to include four additional questions that assess exposure to community violence in addition to questions on household dysfunction and abuse/neglect, as well as aggregate-level responding [20,26]. This modification has been shown to produce significantly higher detection rates of caregiver ACE scores, likely due to increased comfort and privacy in not having to disclose individual traumatic experiences [20]. Higher scores on the ACE measure indicate greater ACE exposure. An ACE score of 4 or higher has been shown to differentiate individuals who are at “high risk” for the detrimental physical and social effects of exposure to toxic stress [12,31]. 

Frequency counts and percentages were generated for categorical variables. Medians (md) and interquartile ranges (IQR) were summarized for continuous variables, and bivariate correlations were conducted to examine associations between variables. Given that caregiver ratings of acceptability and ADI were not normally distributed across clinic populations, Mann–Whitney tests were used to compare differences across groups. Data analysis was conducted using SPSS (Version 28) and SAS (Version 9.4).

## 3. Results

The overwhelming majority of caregivers reported acceptability or neutrality related to being asked about their childhood experiences (95.0%) and recent emotional distress (96.0%), including those who chose not to answer ACE questions (n = 99). Ratings of acceptability related to childhood experiences did not differ across clinic populations (*p* > 0.05). Caregiver ratings of acceptability related to distress were significantly lower for caregivers in the SCD clinic (md = 4, IQR = 3–4) compared to the pain clinic (md = 4, IQR = 4–5, *p* ≤ 0.05), although most caregivers in the SCD clinic still reported acceptability (64.0%) or neutrality (28.0%). Of note, socioeconomic disadvantage based on ADI scores was higher for families in the SCD clinic (md = 9, IQR = 7–10) compared to the pain clinic (md = 3, IQR = 2–6.5, *p* ≤ 0.0001). 

ACE scores did not differ across clinic settings (*p* > 0.05). Consistent with acceptability ratings, the response rate was high, as only one caregiver chose not to answer ACE questions. Four other caregivers chose not to answer questions related to abuse/neglect, but answered the questions focused on household dysfunction and community violence. Given the correlative nature of toxic stressors [18], mean imputation was conducted for missing data. In Table 2, results indicated that 38.0% of caregivers endorsed an ACE score of 4 or higher on the 14-item scale. When examining the 10 original ACEs, 31.0% of caregivers endorsed a score of 4 or higher, indicating high risk for toxic stress. Caregivers reported a median recent distress score of 2 (IQR 0–5) and resilience score of 7 (IQR 6–8). 

Bivariate correlations were conducted to examine the associations between caregiver ratings of acceptability and other caregiver/family variables, including socioeconomic disadvantage. As shown in Table 3, caregiver ratings of acceptability related to distress were negatively correlated with ADI scores (r = −0.22, *p* ≤ 0.05), indicating that caregivers with greater levels of socioeconomic disadvantage reported lower levels of acceptability. Similarly, the relation between caregiver ratings of acceptability related to childhood experiences and ADI approached significance (r = −0.20, *p* ≤ 0.10). Caregiver ratings of acceptability related to distress were positively correlated with caregiver ratings of acceptability related to childhood experiences (r = 0.79, *p* ≤ 0.001). The relation between caregiver ratings of acceptability related to distress and resilience approached significance (r = 0.19, *p* ≤ 0.10), indicating that caregivers who perceived themselves to be more resilient reported higher acceptability.

## 4. Discussion

The overall objective of this study was to understand the feasibility and acceptability of assessing distress, ACEs, and resilience in pediatric subspecialty care settings. Results suggest that family caregivers of patients in pediatric subspecialty clinics were open to being asked about their childhood experiences and recent emotional stress, although ratings of acceptability varied with level of socioeconomic status. Although caution should be used when interpreting trend findings, results suggest that caregiver perceptions of acceptability may also relate to caregiver resilience. Overall, caregivers in these clinics also perceived themselves to be resilient in the face of adversity. 

Caregivers in the SCD clinic reported high levels of acceptability or neutrality related to asking about recent distress, but their ratings were lower than caregivers in the pain clinic, which may be due to underlying socioeconomic factors. SCD is a hereditary disorder that primarily impacts individuals of African, Mediterranean, and Latin American descent [32], and families of youth with SCD face a disproportionate number of psychosocial stressors in the U.S. [22,33]. This likely contributed to the differences observed in the current study. Interestingly, research suggests that caregivers, including those with lower SES, are open to screening for SDOHs in the pediatric setting (e.g., [24,25]). However, caregivers have noted the importance of the context in which this is done, such as screening with a trusted provider/setting, providing caregivers with psychoeducation about the connection between SDOHs and child health, and facilitating referrals to community services [34]. It is possible that caregivers in the current study, especially those with more psychosocial stressors, would have benefitted from SDOH screening (e.g., housing, food insecurity, transportation barriers, etc.) and connection to resources, given that those barriers to care may be more salient and driving emotional distress.

The importance of obtaining information on caregivers’ childhood experiences is underscored by the data in the current study. Caregivers reported extremely high levels of ACEs. Nearly 40% reported an ACE score of 4 or higher, as compared to 11% of caregivers in a general pediatric clinic using the same 14-item measure [20]. Caregivers also reported high levels of recent distress, consistent with research examining parental stress amongst youth with chronic illness [7]. The high rate of ACE reporting underscores one unique benefit of the measure used in this study. Specifically, the measure allows caregivers to report on the number of ACEs across four domains, but does not require them to describe which specific situations they had experienced. Though in this instance the data was being used as part of a research study, there is a potential clinical corollary suggesting that asking more broadly about experiences without disclosure of specific, private information may be beneficial when gathering information about sensitive topics. 

Limitations of the current study include an underrepresentation of fathers and non-English-speaking caregivers. The lack of non-English-speaking caregivers is important to address in future work given the unique stressors faced by immigrant and refugee families in pediatric healthcare settings. In addition, the use of a single site design and convenience sample precludes us from understanding whether the findings are generalizable and whether those caregivers who chose not to answer specific questions were uniquely averse to being asked about sensitive topics. Finally, the use of ADI to assess socioeconomic status is an imprecise and imperfect measure. As noted above, asking more direct questions about SDOHs along with the constructs evaluated in this study may provide a clearer picture of additional stressors being faced by caregivers, patients, and families. 

Anecdotally, when administering surveys for this study, some families asked about why this data was being collected and what the potential follow-up would be. These questions highlight the importance of providing rationale and appropriate psychoeducation when asking about sensitive topics. Future studies should aim to provide more psychoeducation about the potential impact of caregiver well-being on child health, as well as continue to support and bolster efforts to connect families to the kinds of resources and interventions that may provide additional support. More research is needed with a larger population of youth and families, especially when considering the potential impact of inquiry.

In conclusion, results of our study suggest that caregivers broadly found asking about their current distress, ACEs, and resilience acceptable within two pediatric subspecialty clinics. Screening these constructs in the pediatric subspecialty clinic setting offers unique opportunities for clinicians to address families’ psychosocial needs and foster resilience and positive relationships. This kind of compassionate surveillance provides information that could be used to improve referrals to mental health care and other psychosocial supports. 

## Figures and Tables

**Table 1:** Caregiver and Child Demographics.

Caregiver Factors		Child Factors	
Age (years), Median (IQR)	39 (33–43)	Age (years), Median (IQR)	12 (8–15)
Sex, n (%)		Sex, n (%)	
Female	93 (93.0)	Female	53 (53.0)
Male	7 (7.0)	Male	47 (47.0)
Caregiver status, n (%)		Clinic setting, n (%)	
Mother	91 (91.0)	Sickle cell clinic	50 (50.0)
Father	6 (6.0)	Pain clinic	50 (50.0)
Grandmother	3 (3.0)		
Race, n (%)		Race, n (%)	
African American	53 (53.0)	African American	55 (55.0)
White	41 (41.0)	White	39 (39.0)
American Indian/Alaskan Native	1 (1.0)	American Indian/Alaskan Native	1 (1.0)
Multiethnic	2 (2.0)	Asian	1 (1.0)
Other	1 (1.0)	Multiethnic	3 (3.0)
Declined to answer	2 (2.0)	Declined to answer	1 (1.0)
Ethnicity, n (%) ^+^		Ethnicity, n (%)	
Non-Hispanic	86 (86.0)	Non-Hispanic	82 (82.0)
Hispanic	6 (6.0)	Hispanic	12 (12.0)
Declined to answer	7 (7.0)	Declined to answer	6 (6.0)
ADI state rank, n (%) ^+^			
1–3	32 (32.0)		
4–6	15 (15.0)		
7–10	51 (51.0)		
Marital Status n (%) ^+^			
Not Married	34 (34.0)		
Married	51 (51.0)		
Cohabitating	5 (5.0)		
Separated/Divorced	9 (9.0)		

Note. N = 100 families, including 100 caregivers and 100 youth. ADI = Area Deprivation Index. Higher rankings indicate greater socioeconomic disadvantage on the state level. ^+^ Indicates missing data for some participants.

**Table 2:** Caregiver Study Variables.

Caregiver ratings of acceptability—childhood experiences, n (%) ^+^	
Extremely unacceptable	3 (3.0)
Unacceptable	1 (1.0)
Neutral	25 (25.0)
Acceptable	44 (44.0)
Extremely acceptable	26 (26.0)
Caregiver ratings of acceptability—recent distress ^+^	
Extremely unacceptable	2 (2.0)
Unacceptable	1 (1.0)
Neutral	24 (24.0)
Acceptable	43 (43.0)
Extremely acceptable	29 (29.0)
14-item ACE, Median (IQR) ^+^	2.0 (0.0–7.0)
14-item ACE prevalence, n (%) ^+^	
0	26 (26.0)
1–3	35 (35.0)
≥4	38 (38.0)
10-item ACE, Median (IQR) ^+^	2.0 (0.0–5.0)
10-item ACE prevalence, n (%) ^+^	
0	31 (31.0)
1–3	37 (37.0)
≥4	31 (31.0)
NCCN, Median (IQR)	2.0 (0.0–5.0)
CD-RISC 2, Median (IQR)	7.0 (6.0–8.0)

Note. N = 100 caregivers. ACE = caregiver report of adverse childhood events, NCCN = caregiver report of recent distress, CD-RISC 2 = caregiver rating of self-reported resilience. ^+^ Indicates missing data for some participants.

**Table 3:** Correlations for Caregiver Variables and Socioeconomic Disadvantage.

	ACE-14	ACE-10	CD-RISC 2	NCCN	Acceptability-ACEs	Acceptability-Distress	ADI
ACE-14	-						
ACE-10	0.97 ***	-					
CD-RISC 2	−0.07	−0.06	-				
NCCN	0.46 ***	0.49 ***	−0.20 *	-			
Acceptability-ACEs	−0.12	−0.11	0.08	−0.12	-		
Acceptability-Distress	−0.09	−0.06	0.19 ^+^	−0.03	0.79 ***	-	
ADI	0.21 *	0.16	−0.003	−0.04	−0.20 ^+^	−0.22 *	-

Note. N = 100 families, including 100 caregivers and 100 youth. ACE-14 = caregiver report of adverse childhood events (ACEs) 14-item measure, ACE-10 = ACEs 10-item measure, CD-RISC 2 = caregiver rating of self-reported resilience, NCCN = caregiver recent emotional distress, ADI = Area Deprivation Index. * *p* ≤ 0.05; *** *p* ≤ 0.001, ^+^ *p* ≤ 0.10.

## Data Availability

Data are available upon request.
